# Machine learning approach for personalized vancomycin steady-state trough concentration prediction: a superior approach over Bayesian population pharmacokinetic model

**DOI:** 10.3389/fphar.2025.1549500

**Published:** 2025-06-12

**Authors:** Ting Hu, Xian Ding, Feifei Han, Zhuoling An

**Affiliations:** Department of Pharmacy, Beijing Chao-Yang Hospital, Capital Medical University, Beijing, China

**Keywords:** vancomycin, trough concentration, random forest, bnp, creatinine clearance, Bayesian PopPK model

## Abstract

**Introduction:**

Appropriate vancomycin trough levels are crucial for ensuring therapeutic efficacy while minimizing toxicity. The aim of this study is to identify clinical factors that influence the steady-state trough concentration of vancomycin and to establish a machine learning model for accurately predicting vancomycin’s steady-state trough concentration.

**Methods:**

This study is a single-center, retrospective, observational investigation involving 546 hospitalized patients who received intravenous vancomycin therapy. A total of 57 clinical indicators were collected from the subjects. Random forest models were constructed and validated using internal and external datasets, with performance compared to a Bayesian PopPK model.

**Results:**

The random forest model incorporated a comprehensive set of clinical indicators, including creatinine clearance, C-reactive protein (CRP), B-type natriuretic peptide (BNP), high-density lipoprotein cholesterol (HDL-C), and daily vancomycin dose, collected 48 hours before steady-state concentration assessment. The random forest regression model achieved correlation coefficients of 0.94 for the training set and 0.81 for the test set, respectively. The random forest classification model demonstrated impressive accuracy rates of 0.99 for the training set and 0.84 for the test set. External validation further confirmed the model’s generalization capabilities, with a predictive accuracy of 0.83, surpassing the Bayesian PopPK model’s 0.57 accuracy.

**Discussion:**

This study presents a robust random forest model that predicts vancomycin steady-state trough concentrations with high accuracy, offering a significant advantage over existing Bayesian PopPK model. By integrating diverse clinical indicators, the model supports personalized medicine approaches and has the potential to improve clinical outcomes by facilitating more precise dosing strategies.

## Introduction

Vancomycin is a glycopeptide antibiotic originally derived from *Streptomyces* orientalis (Cairns et al., 2023; Levine, 2006). It is a cornerstone in the treatment of serious gram-positive bacterial infections, particularly for strains resistant to other antibiotics such as methicillin-resistant *Staphylococcus aureus* (MRSA) (Rybak, 2006; Nailor and Sobel, 2009). The clinical use of vancomycin is often reserved for severe infections or when other antibiotics are ineffective or contraindicated (Álvarez et al., 2016; Rybak et al., 2020a). It can be used for a variety of indications, including skin and soft tissue infections, bone and joint infections, lower respiratory tract infections, and for the treatment of endocarditis and bacteremia (Rybak et al., 2020a; Rybak M. et al., 2009). Vancomycin is typically administered via intravenous infusion for systemic infections, as it is not significantly absorbed systemically when taken orally (Wahby et al., 2021; Kasia et al., 2005). Vancomycin is primarily eliminated by kidney, and dosage adjustments are necessary for patients with impaired renal function (Rybak, 2006; Pai et al., 2014). The adverse effects of vancomycin include nephrotoxicity, ototoxicity, and hypersensitivity reactions, necessitating careful patient monitoring during treatment (Lestner et al., 2016; Rybak M. J. et al., 2009).

As recommended by guidelines, monitoring steady-state trough concentration of serum vancomycin levels is essential to ensure therapeutic efficacy and to minimize the risk of toxicity (He et al., 2020; Matsumoto et al., 2022). Vancomycin steady-state trough concentrations is crucial as it helps in achieving optimal pharmacokinetic/pharmacodynamic (PK/PD) targets, such as the area under the curve to minimum inhibitory concentration ratio (AUC/MIC), which is associated with treatment success (Neely et al., 2018; Prybylski, 2017). Adequate steady-state trough levels (10–20 μg/mL) are recommended for infections like MRSA bacteremia, as they improve clinical outcomes and reduce the risk of treatment failure (Matsumoto et al., 2022; Rybak et al., 2020b). Furthermore, monitoring is significant in adjusting dosing regimens to prevent nephrotoxicity, a potential adverse effect, especially in patients with compromised renal function or those receiving concurrent nephrotoxic medications (Rybak et al., 2020a; He et al., 2020). Thus, therapeutic drug monitoring of vancomycin is essential for personalized medicine approaches, ensuring that each patient receives the most effective and safe treatment possible.

Clinical dosage of vancomycin is usually tailored to the patient’s condition, including renal function, age and body weight, with serum trough levels closely monitored to ensure efficacy and safety (Drennan et al., 2019; Vandecasteele et al., 2013). However, according to the recommended dose calculation method, a significant proportion of patients still struggle to achieve a satisfactory serum concentration range in the initial trough concentration monitoring after reaching steady-state. Though it was used according to the vancomycin guidelines, over 50% of the hospitalized patients did not have vancomycin steady-state trough concentrations within the target range (10–20 μg/mL) after reaching steady-state from the clinical practice of our hospital. This indicates that determining the dosage of vancomycin based solely on factors such as patient age, weight, renal function, severity of infection, and pathogen sensitivity is not sufficient to ensure satisfactory serum concentrations of vancomycin for most patients.

Predicting vancomycin trough concentrations early before treatment is crucial for timely clinical decision-making and patient safety. Current models for predicting vancomycin’s steady-state trough concentrations primarily rely on Bayesian PopPK model (Chen et al., 2018; Berthaud et al., 2019; Hui et al., 2022; Ohnishi et al., 2005; Wrishko et al., 2000). These models typically incorporate clinical indicators such as creatinine levels, and administration dose to enhance the precision of dosage adjustments. Bayesian models for vancomycin steady-state trough concentration prediction offer personalized dosing tailored to individual patient characteristics, enhancing treatment efficacy and safety. However, the complexity of patient physiology and pathophysiology may not be fully captured, leading to potential inaccuracies in predictions. Additionally, the reliance on certain assumptions in the Bayesian framework can also introduce bias, affecting the model’s generalizability across different patient populations. The determination of the dosage of vancomycin requires more in-depth research to identify other clinical and individual factors that affect its blood concentration. Formulating a more scientific dosage regimen for vancomycin is of great value for enhancing the scientific, effective, and safe application of vancomycin in clinical practice.

Therefore, the core objective of this study is to utilize machine learning techniques to deeply analyze and understand patients’ physiological, pathogen information, as well as the pharmacokinetic and pharmacodynamic characteristics of vancomycin. We aim to identify clinical factors, laboratory test indicators, and pathological factors that affect vancomycin blood concentrations. Leveraging machine learning technology, we will integrate clinical and pathological factors significantly related to vancomycin blood concentrations to build a calculation model for the initial dosage of vancomycin. This is expected to provide personalized medication guidance for patients, thereby maximizing therapeutic effects and minimizing the risk of drug-related toxic side effects and antibiotic resistance.

## Methods

### Study design and population

This is a single-center retrospective observational study. This study enrolled 546 inpatients who were treated with vancomycin at Beijing Chaoyang Hospital from January 2022 to February 2024. Newborns and pregnant women were excluded. Vancomycin dosage should meet criteria for PK-guided dosing recommendation according to 2009 ASHP vancomycin TDM guideline (Rybak M. J. et al., 2009). The participants must receive at least 48 h of intravenous vancomycin therapy and underwent vancomycin trough concentration monitoring at the hospital’s therapeutic drug monitoring laboratory after reaching steady-state blood concentrations, with available steady-state trough concentration information. Additionally, we excluded those who received non-intravenous routs of vancomycin.

### Ethical approval

All procedures performed in this study involving human participants were conducted in accordance with the Declaration of Helsinki and was approved by the ethics committee of Beijing Chao-Yang Hospital (Ethics Code: 2024-Research-502). According to the Regulations of the ethics committee of Beijing Chao-Yang Hospital on Exemption of informed Consent, those who plan to use the medical records obtained in previous clinical treatment in the study of human subjects can be exempted from informed consent. This study met the conditions for an exemption from informed consent and was approved by the Ethics Committee for an exemption from informed consent. All the clinical information were collected from the hospital’s case system in accordance with ethical guidelines.

### Clinical laboratory testing and data collection

Demographic information, including age, gender, height, weight, blood pressure and disease information, was recorded for each of the subject. The first steady-state trough concentrations of vancomycin were collected for all the enrolled subjects. The clinical laboratory test results for clinical indicators, including blood biochemistry, coagulation function, and complete blood count, were obtained from the clinical laboratory reports of the hospital’s laboratory department for all included patients. These tests were conducted 48 h before the first steady-state trough concentration measurement.

### Grouping of subjects by vancomycin steady-state trough concentration

According to the first steady-state trough concentrations of vancomycin, the subjects were divided to three different groups, including low trough group with steady-state trough levels less than 10 μg/mL (N = 154), appropriate trough group with steady-state trough levels ranged between 10 and 20 μg/mL (N = 234), and excessive trough group with steady-state trough levels larger than 20 μg/mL (N = 158).

### Data processing and statistical analysis

Two-sided unpaired Welch’s t-test was performed for each pair of comparing groups and adjusted P values were calculated using Benjamini and Hochberg correction. IBM SPSS 21 (Armonk, New York, United States) was used for Spearman’s correlation, with P < 0.05 considered as statistically significant. Scientific Platform Serving for Statistics Professional (SPSSPRO) developed by Zhongyan Technology Co., LTD., was used for the establishment and validation of machine learning models (Suzhou, Jiangsu, China) (Author Anonymous, 2022). SIMCA 14.1 (Umetrics AB, Umeå, Sweden) was employed for multivariate statistical analysis, including principal component analysis (PCA) and Partial least squares discriminant analysis (PLS-DA). Graphpad Prim 8 was used for receiver operating characteristic (ROC) and histogram analysis. Vancomycin Calculator (https://clincalc.com/vancomycin/) was used for prediction of vancomycin steady-state trough concentrations based on Bayesian population pharmacokinetic (PopPK) model (Kane, 2024). Linear regression analysis was performed by Hiplot Pro (https://hiplot.com.cn/), a comprehensive web service for biomedical data analysis and visualization. For missing values, impute them by using the average values specific to their corresponding trough concentration level groups. Log transformation is often used in machine learning to reduce skewness, stabilize variance, and linearize relationships, making it easier for models to learn effective patterns from the data. For machine learning model development of this study, logarithmic transformations were performed on numerical variables.

## Results

### Baseline characteristics of the participants

**FIGURE 1:**
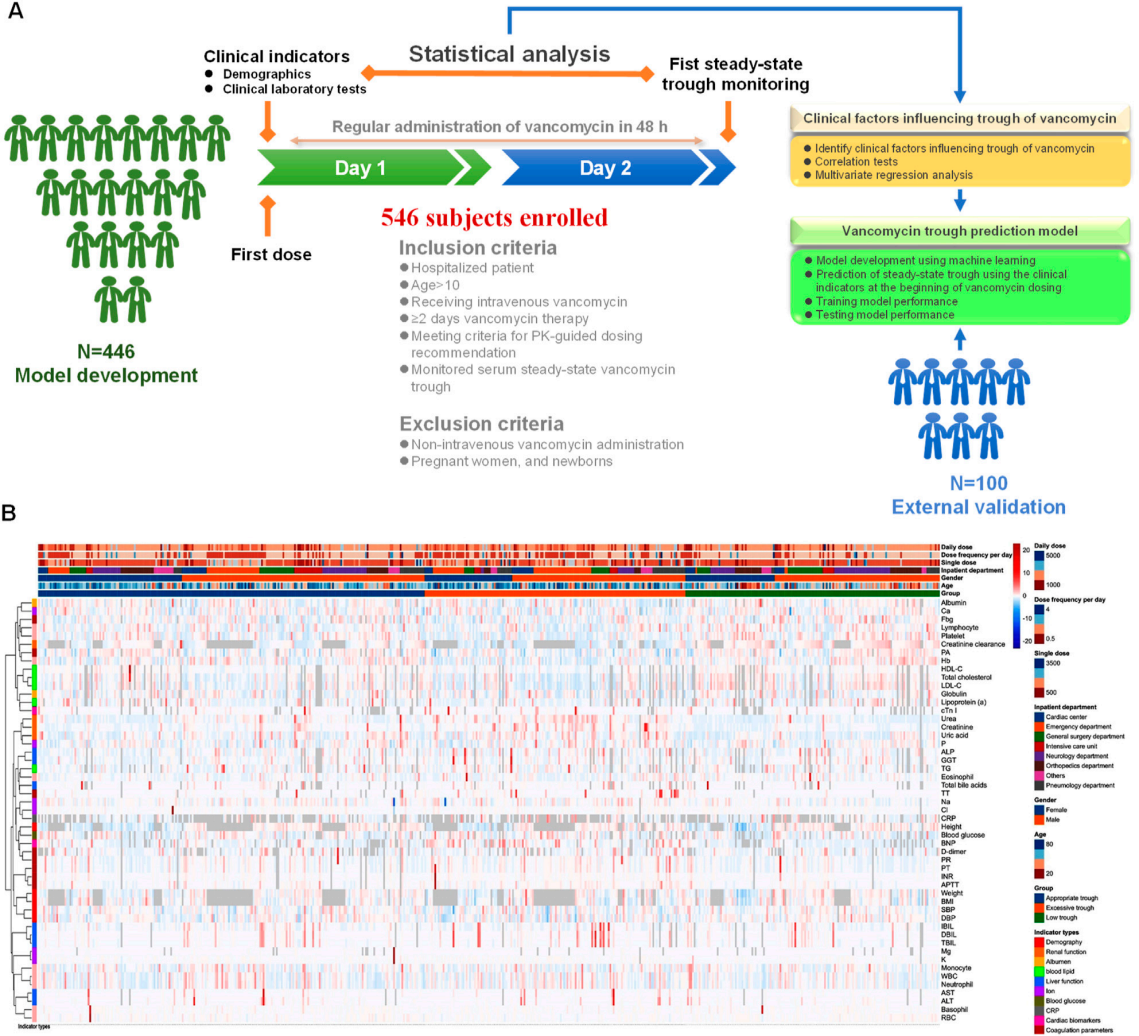
Patient enrollment, study design, and clinical indicators. **(A)** The schematic summary of the study design, subject enrollment, clinical indicator collection and statistical analysis. **(B)** Heatmap of clinical indicators for low trough, appropriate trough and excessive trough groups.

The study design was summarized in Figure 1A. A total of 546 hospitalized patients who met the inclusion and exclusion criteria were included in the final statistical analysis. Baseline characteristics of the participants with different vancomycin trough levels were shown in Table 1 and heatmap of Figure 1B. There were no statistically significant differences in body weight, height and body mass index (BMI) among the three groups with different steady-state trough concentrations of vancomycin. While six variables were significantly difference among the three different groups, including age, inpatient department, creatinine clearance, DBP, daily dose of vancomycin, and steady-state trough. The low trough concentration group had a lower median age, while the high concentration group consisted of older patients. Subjects in low trough group were primarily from the surgical system. While subjects in excessive trough group were mainly from Emergency (34%) and Pulmonology (19%). Patients with lower creatinine clearance were more likely to exhibit supratherapeutic trough concentrations, a fact that is evident and widely reported in the literature. DBP progressively decreased across the low trough, appropriate trough, and excessive trough groups. The median daily dose of vancomycin was 2,000 mg in all the three groups. However, there was still a statistical difference (P = 0.012) in the daily dose among the three groups. Dose frequency per day was also different among the three groups. The median steady-state trough concentrations of vancomycin were 7, 15, and 28 µg/mL in low trough group, appropriate trough group, and excessive trough group, respectively.

**TABLE 1:** Baseline characteristics of the subjects with different vancomycin trough levels.

Variable	Overall N = 546^a^	Low trough N = 154	Appropriate trough N = 234	Excessive trough N = 158	P value^b^
Gender, n (%)					0.75
Female	194 (36)	54 (35)	87 (37)	53 (34)	
Male	352 (64)	100 (65)	147 (63)	105 (66)	
Age, Median (IQR)	62 (49–72)	54 (38–62)	65 (53–73)	67 (57–77)	<0.001
Weight, Median (IQR)	66 (60–75)	70 (60–77)	67 (60–75)	64 (58–75)	0.43
Bedridden	144	23	67	54	
Height, Median (IQR)	168 (160–173)	169 (162–174)	168 (160–173)	170 (160–173)	0.69
Bedridden	144	23	67	54	
BMI, Median (IQR)	24.1 (21.8–26.4)	24.2 (22.1–27.0)	24.2 (22.0–26.5)	23.5 (21.5–25.7)	0.28
Bedridden	144	23	67	54	
Inpatient department, n (%)					<0.001
Cardiac center	51 (9.3)	12 (7.8)	21 (9.0)	18 (11)	
Emergency department	102 (19)	3 (1.9)	45 (19)	54 (34)	
General surgery department	77 (14)	29 (19)	31 (13)	17 (11)	
Intensive care unit	37 (6.8)	7 (4.5)	21 (9.0)	9 (5.7)	
Neurology department	105 (19)	49 (32)	44 (19)	12 (7.6)	
Orthopedics department	76 (14)	35 (23)	33 (14)	8 (5.1)	
Pneumology department	59 (11)	10 (6.5)	20 (8.5)	29 (18)	
Others	39 (7.1)	9 (5.8)	19 (8.1)	11 (7.0)	
Creatinine clearance, Median (IQR)	100 (57–136)	134 (110–178)	96 (69–123)	52 (38–95)	<0.001
NA	146	24	67	55	
SBP, Median (IQR)	130 (120–140)	130 (121–139)	130 (120–140)	128 (114–142)	0.65
NA	1	1	0	0	
DBP, Median (IQR)	78 (69–84)	80 (71–85)	78 (70–84)	72 (61–84)	<0.001
NA	1	1	0	0	
Daily dose (mg), Median (IQR)	2,000 (1,500–2,000)	2,000 (2,000–2,000)	2,000 (1,500–2,000)	2,000 (1,500–2,000)	0.012
Dose frequency per day, n (%)					
0.33	1 (0.2)	0 (0)	1 (0.4)	0 (0)	
1	188 (34)	26 (17)	83 (35)	79 (50)	
1.5	1 (0.2)	1 (0.6)	0 (0)	0 (0)	
2	326 (60)	119 (77)	138 (59)	69 (44)	
3	25 (4.6)	6 (3.9)	11 (4.7)	8 (5.1)	
4	5 (0.9)	2 (1.3)	1 (0.4)	2 (1.3)	
Steady-state trough, Median (IQR)	15 (9–21)	7 (5–8)	15 (12–17)	28 (24–35)	<0.001

^a^ , N (%); Median (IQR).

^b^ , P-values are calculated through hypothesis testing. For continuous variables, the distribution is first assessed using the Shapiro-Wilk test. Normally distributed data are analyzed using two-sided Student's t-tests. For non-parametric data, the Mann-Whitney U test is applied. For categorical variables, P-values are computed using the chi-square test.

Abbreviation: IQR, interquartile range; NA, not available; SBP, systolic blood pressure; DBP, diastolic blood pressure.

### Clinical laboratory test results

Clinical laboratory test results for 45 clinical indicators, including blood biochemistry, coagulation function, and complete blood count, measured 48 h before the first steady-state trough concentration measurement are presented in Table 2 and visualized in the heatmap of Figure 1B. Blood biochemical indicators such as albumin, total cholesterol, HDL-C, LDL-C, direct bilirubin (DBIL), total bile acids, urea, creatinine, uric acid, sodium (Na), blood glucose, C-reactive protein (CRP), cardiac troponin I (cTn I), and B-type natriuretic peptide (BNP) showed statistically significant differences among the low trough group, the appropriate trough group, and the excessive trough group. Kidney function related indicators, including urea, creatinine, and uric acid, showed the same trend across the three different groups, with the excessive trough group having the highest levels, followed by the appropriate trough group and low trough group. Blood cholesterol indicators, including the total cholesterol, HDL-C, and LDL-C, also exhibited the same trend across the three different groups with their significance P values less than 0.001. Highest values of blood cholesterol indicators occurred in the low trough group, followed by the appropriate trough group and excessive trough group. BNP and cTn I, which are biomarkers used in clinical practice for the assessment of cardiac function and myocardial damage, were also significantly difference (P < 0.001) among the three groups. The elevation of these cardiac injury biomarker levels increases the risk of excessively high vancomycin trough concentrations. Conversely, levels that were too low can result in suboptimal trough concentrations that do not achieve the effective therapeutic range.

**TABLE 2:** Clinical laboratory tests of the subjects with different vancomycin trough levels.

Variable	Overall N = 546	Low trough N = 154	Appropriate trough N = 234	Excessive trough N = 158	P value
Albumin (g/L), Median (IQR)	34.2 (30.6–38.0)	34.8 (32.3–37.5)	34.5 (30.5–38.2)	32.5 (29.2–36.6)	0.003
NA	17	7	5	5	
Globulin (g/L), Median (IQR)	24.0 (20.6–27.3)	23.7 (20.2–26.7)	24.3 (20.8–27.0)	23.8 (19.5–28.3)	0.61
NA	46	15	19	12	
Total cholesterol (mmol/L), Median (IQR)	3.15 (2.31–3.97)	3.72 (2.95–4.20)	3.19 (2.34–3.74)	2.62 (1.95–3.61)	<0.001
NA	57	21	22	14	
HDL-C (mmol/L), Median (IQR)	0.66 (0.43–0.88)	0.76 (0.60–0.97)	0.70 (0.47–0.92)	0.49 (0.34–0.71)	<0.001
NA	57	21	22	14	
LDL-C (mmol/L), Median (IQR)	1.86 (1.18–2.62)	2.39 (1.75–3.04)	1.85 (1.27–2.50)	1.39 (0.89–2.19)	<0.001
NA	58	21	23	14	
TG (mmol/L), Median (IQR)	1.27 (0.90–1.81)	1.28 (0.91–1.80)	1.20 (0.86–1.68)	1.34 (0.95–1.97)	0.12
NA	57	21	22	14	
Lipoprotein (a) (mg/dL), Median (IQR)	13 (7–27)	15 (7–33)	14 (7–26)	12 (6–21)	0.05
NA	61	22	24	15	
AST (U/L), Median (IQR)	31 (21–55)	29 (21–48)	30 (20–53)	33 (24–58)	0.12
NA	9	4	4	1	
ALT (U/L), Median (IQR)	29 (17–60)	31 (18–66)	28 (17–60)	28 (16–54)	0.41
NA	9	4	4	1	
ALP (U/L), Median (IQR)	88 (64–128)	84 (63–126)	84 (65–117)	91 (62–140)	0.5
NA	42	14	16	12	
GGT (U/L), Median (IQR)	44 (22–96)	47 (25–100)	40 (20–86)	44 (23–106)	0.42
NA	42	14	16	12	
DBIL (µmol/L), Median (IQR)	6 (4–11)	5 (3–8)	6 (4–10)	6 (4–14)	0.021
NA	16	7	4	5	
IBIL (µmol/L), Median (IQR)	6 (4–10)	6 (4–10)	6 (4–10)	6 (4–9)	0.25
NA	16	7	4	5	
TBIL (µmol/L), Median (IQR)	12 (8–20)	12 (8–18)	12 (8–20)	12 (8–24)	0.54
NA	16	7	4	5	
Total bile acid (µmol/L), Median (IQR)	5 (3–9)	4 (3–7)	5 (2–8)	6 (3–13)	0.037
NA	48	15	21	12	
Urea (mmol/L), Median (IQR)	8 (5–13)	5 (4–7)	8 (5–12)	13 (9–18)	<0.001
NA	2	1	0	1	
Creatinine (µmol/L), Median (IQR)	62 (46–93)	48 (38–67)	61 (46–83)	93 (62–167)	<0.001
NA	2	1	0	1	
Uric acid (µmol/L), Median (IQR)	163 (106–254)	135 (89–199)	164 (108–248)	204 (132–302)	<0.001
NA	2	1	0	1	
Ca (mmol/L), Median (IQR)	2.02 (1.91–2.13)	2.05 (1.93–2.13)	2.03 (1.91–2.14)	2.00 (1.88–2.12)	0.14
NA	2	1	0	1	
P (mmol/L), Median (IQR)	0.94 (0.74–1.15)	0.95 (0.75–1.16)	0.92 (0.73–1.15)	0.94 (0.76–1.15)	0.58
NA	2	1	0	1	
Mg (mmol/L), Median (IQR)	0.83 (0.74–0.90)	0.83 (0.76–0.88)	0.84 (0.75–0.90)	0.81 (0.73–0.91)	0.45
NA	29	12	10	7	
Na (mmol/L), Median (IQR)	139 (136–144)	138 (136–141)	140 (136–144)	140 (136–145)	0.026
NA	2	1	0	1	
K (mmol/L), Median (IQR)	4.10 (3.70–4.40)	4.00 (3.80–4.30)	4.10 (3.70–4.40)	4.10 (3.70–4.50)	0.39
NA	2	1	0	1	
Cl (mmol/L), Median (IQR)	104 (101–108)	103 (101–107)	104 (100–108)	104 (100–109)	0.45
NA	2	1	0	1	
Blood glucose (mmol/L), Median (IQR)	7.7 (5.4–10.6)	6.7 (5.0–9.5)	7.7 (5.6–10.5)	8.6 (6.3–11.1)	0.002
NA	56	19	22	15	
CRP (mg/L), Median (IQR)	35 (12–77)	34 (12–83)	30 (10–62)	50 (22–104)	0.025
NA	244	54	106	84	
cTn I (ng/mL), Median (IQR)	30 (4–170)	10 (0–64)	20 (3–138)	70 (17–420)	<0.001
NA	58	25	28	5	
BNP (pg/mL), Median (IQR)	176 (58–600)	73 (31–204)	160 (56–536)	492 (153–1,262)	<0.001
NA	66	26	30	10	
PT (s), Median (IQR)	13.30 (12.40–14.55)	12.85 (12.10–13.93)	13.30 (12.40–14.30)	13.90 (13.00–15.50)	<0.001
NA	11	2	9	0	
PA (%), Median (IQR)	76 (67–84)	80 (72–86)	77 (69–85)	71 (62–79)	<0.001
NA	11	2	9	0	
PR, Median (IQR)	1.17 (1.08–1.27)	1.12 (1.06–1.22)	1.16 (1.08–1.25)	1.23 (1.13–1.36)	<0.001
NA	11	2	9	0	
INR, Median (IQR)	1.17 (1.08–1.29)	1.12 (1.05–1.23)	1.16 (1.07–1.26)	1.24 (1.13–1.37)	<0.001
NA	11	2	9	0	
APTT (s), Median (IQR)	31 (28–36)	29 (26–34)	30 (27–35)	35 (30–42)	<0.001
NA	11	2	9	0	
Fbg (mg/dL), Median (IQR)	389 (269–487)	411 (297–540)	392 (272–488)	341 (250–448)	0.002
NA	11	2	9	0	
TT (s), Median (IQR)	16.4 (15.4–17.7)	16.0 (15.1–16.9)	16.4 (15.4–17.8)	17.1 (16.0–18.8)	<0.001
NA	11	2	9	0	
D-dimer (FEU), Median (IQR)	3 (2–7)	3 (1–6)	3 (2–7)	4 (2–7)	0.36
NA	75	18	39	18	
WBC (10^9/L), Median (IQR)	9.6 (6.7–13.8)	9.4 (6.5–13.2)	9.5 (6.6–13.7)	10.5 (7.0–14.8)	0.29
Neutrophil (10^9/L), Median (IQR)	7.9 (5.1–11.7)	7.7 (4.9–10.8)	7.8 (5.0–11.4)	8.9 (5.6–12.8)	0.16
Lymphocyte (10^9/L), Median (IQR)	0.94 (0.61–1.39)	1.07 (0.75–1.51)	0.89 (0.61–1.38)	0.86 (0.53–1.28)	0.004
Monocyte (10^9/L), Median (IQR)	0.48 (0.31–0.72)	0.52 (0.37–0.72)	0.46 (0.30–0.71)	0.49 (0.29–0.72)	0.22
Eosinophil (10^9/L), Median (IQR)	0.08 (0.01–0.18)	0.11 (0.03–0.21)	0.07 (0.01–0.16)	0.07 (0.01–0.21)	0.035
Basophil (10^9/L), Median (IQR)	0.020 (0.010–0.040)	0.030 (0.020–0.040)	0.020 (0.010–0.048)	0.020 (0.010–0.040)	0.23
RBC (10^12/L), Median (IQR)	2.91 (2.45–3.47)	3.17 (2.66–3.56)	3.00 (2.48–3.50)	2.63 (2.25–3.14)	<0.001
Hb (g/L), Median (IQR)	89 (75–104)	95 (81–109)	91 (77–107)	80 (69–94)	<0.001
Platelet (10^9/L), Median (IQR)	190 (105–280)	250 (161–331)	196 (114–286)	127 (85–223)	<0.001

Abbreviation: HDL-C, high-density lipoprotein cholesterol; LDL-C, low-density lipoprotein cholesterol; TG, triglycerides; AST, aspartate aminotransferase; ALT, alanine aminotransferase; ALP, alkaline phosphatase; GGT, gamma-glutamyl transferase; DBIL, direct bilirubin; IBIL, indirect bilirubin; TBIL, total bilirubin; Ca, calcium; P, phosphorus; Mg, magnesium; Na, sodium; K, potassium; Cl, chloride; CRP, C-reactive protein; cTn I, cardiac troponin I; BNP, B-type natriuretic peptide; PT, prothrombin time; PA, protein C activity; PR, partial thromboplastin ratio; INR, international normalized ratio; APTT, activated partial thromboplastin time; Fbg, fibrinogen; TT, thrombin time; D-dimer, D-dimer; WBC, white blood cell count; RBC, red blood cell count; Hb, hemoglobin.

As shown in Table 2, coagulation function related indicators, including PT (prothrombin time), PA (prothrombin activity), PR (prothrombin time ratio), INR (international normalized ratio), APTT (activated partial thromboplastin time), Fbg (fibrinogen) and TT (thrombin time), were all significantly difference among low trough group, appropriate trough group, and excessive trough group. Complete blood count test indicators, including lymphocyte, eosinophil, RBC, Hb, and platelet, were significantly difference among these three groups. The results indicated that the steady-state trough concentration levels of vancomycin were not only closely related to kidney function but also significantly correlated with cholesterol indicators, cardiac biomarkers, blood glucose, coagulation parameters, and hematological indices.

### Correlation between vancomycin steady-state trough concentration and clinical indicators

**FIGURE 2:**
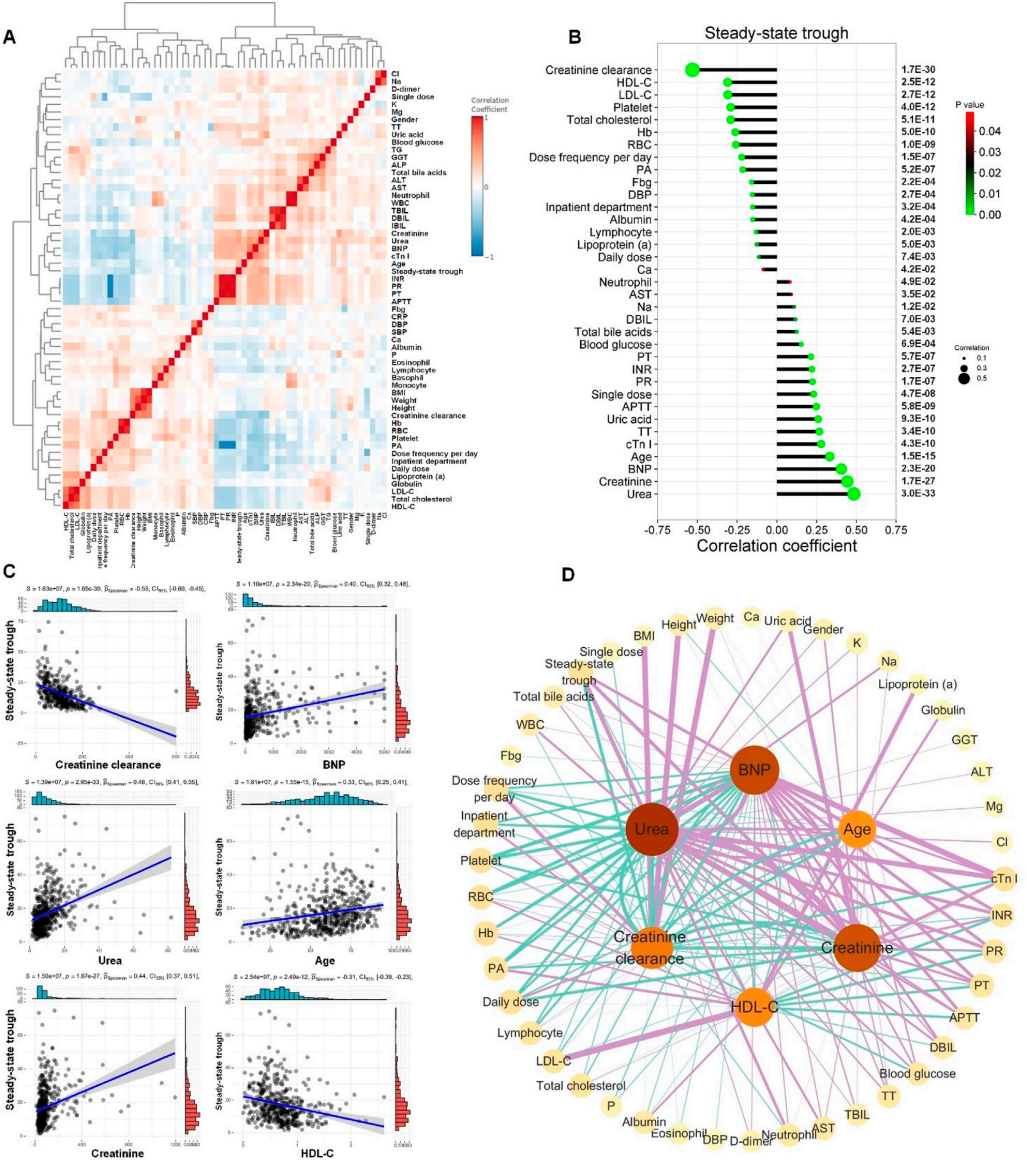
Correlation between vancomycin steady-state trough concentration and clinical indicators. **(A)** Correlations of clinical phenotypes by Spearman’s rank correlation analysis. **(B)** Correlation coefficient lollipop chart exhibited the significant correlations (P < 0.05) between vancomycin steady-state trough concentration and the other clinical indicators. The Y-axis on the right is the Spearman’s correlation P-value. **(C)** The linear regression scatter plots for the six clinical indicators with the most significant correlation to vancomycin steady-state trough concentrations. **(D)** Spearman’s correlation network diagram among creatinine-clearance, urea, creatinine, BNP, age, HDL-C and the other clinical indicators. The figure only displayed correlations with P values less than 0.05. Green lines represented significant negative correlations, while purple lines indicated significant positive correlations. The thicker the line, the stronger the correlation.

Correlation analysis helps to reveal clinical indicators associated with the steady-state trough concentrations of vancomycin. Spearman’s rank correlation analysis was conducted to study the correlation ships between vancomycin steady-state trough concentration and clinical indicators. To visualize the correlations among all the clinical phenotypes, correlation coefficient from Spearman’s rank analysis was presented as a heatmap shown in Figure 2A. The interrelationships among various clinical indicators were intricate, reflecting the multifactorial influences on vancomycin treatment response and patient prognosis.

Significant correlations (P < 0.05) between vancomycin steady-state trough concentration and the other clinical indicators were exhibited in Figure 2B. The top five clinical indicators that showed the most significant positive correlation with vancomycin steady-state trough concentration were creatinine clearance, HDL-C, LDL-C, platelet, and total cholesterol. The top five clinical indicators that exhibited the most significant negative correlation with vancomycin steady-state trough concentrations were ranked as urea, creatinine, BNP, age and cTn I. The linear regression scatter plots for the six clinical indicators with the most significant correlation to vancomycin steady-state trough concentrations are shown in Figure 2C, which were creatinine clearance, urea, creatinine, BNP, age, and HDL-C. Significant correlations (P < 0.05) among these six indicators and the other clinical indicators were shown in Figure 2D. The above results of the Spearman’s rank correlation analysis indicated that the steady-state trough concentration of vancomycin was not only closely related to kidney function but also significantly correlated with serum markers of cardiac function and cholesterol-related lipid indicators.

### Screening of clinical indicators related to the grouping of vancomycin steady-state trough concentrations

**FIGURE 3:**
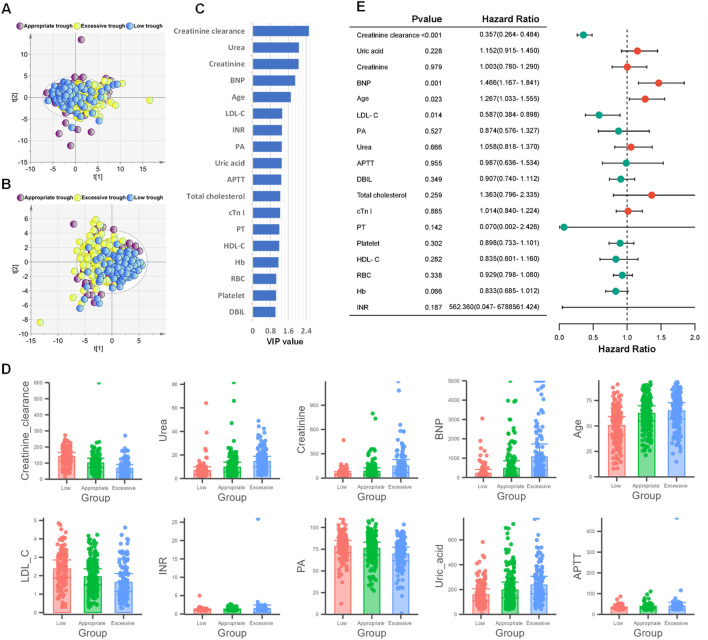
Screening of clinical indicators related to the grouping of vancomycin steady-state trough concentrations. **(A)** PCA score plot based on 57 clinical indicators. **(B)** PLS-DA score plot based on 57 clinical indicators. **(C)** The 18 selected clinical indicators by VIP>1 in PLS-DA model. **(D)** Bar chart plots of the top 10 variables with the highest VIP values. **(E)** Forest plot from ordinal logistic regression analysis based on the 18 clinical indicators with VIP>1.

To further investigate whether the steady-state trough concentrations of vancomycin were associated with clinical indicators 48 h before steady-state trough concentration, we utilized principal component analysis (PCA) and partial least squares-discriminant analysis (PLS-DA) to explore and interpret the complex dataset, aiming to identify the intrinsic patterns and distinguish among low, appropriate and excessive trough groups based on their clinical indicators. PCA effectively reduced dimensionality, capturing the major sources of variance within the data. The resulting score plot of PCA illustrated a clear separation between low trough group and excessive trough group (Figure 3A). Following PCA, we conducted PLS-DA to further investigate the discriminatory capabilities of the three groups using the clinical dataset. Unlike PCA, which is an unsupervised method, PLS-DA is a supervised technique that maximizes the variation between groups, thus enhancing the separation in the score plot (Figure 3B). PLS-DA yielded distinct clusters of low trough, appropriate trough, and excessive trough groups, highlighting the discriminatory potential of the selected clinical features.

As shown in Figure 3C, 18 clinical indicators possessed VIP>1. As depicted in Figure 3C, a total of 18 clinical indicators in the PLS-DA model have variable importance in projection (VIP) values exceeding 1, indicating their significant contribution to the model. The levels of the top 10 variables with the highest VIP values were shown in Figure 3D. A further ordinal logistic regression analysis was conducted based on these clinical indictors with VIP>1 (Figure 3E). In the logistic regression analysis, creatinine clearance, BNP, age and LDL-C exhibited p-values less than 0.05, which indicated a strong association between these four predictor variables and the steady-state trough concentrations of vancomycin. The odds ratios (ORs) of creatinine clearance and LDL-C were less than 1, suggesting an inverse relationship with the steady-state trough concentration of vancomycin. Conversely, the ORs of 1.466 and 1.267 indicated that the steady-state trough concentrations were positively correlated with both BNP levels and age.

### Development of vancomycin steady-state trough concentration prediction model utilizing machine learning techniques

The aforementioned research findings indicate that the clinical factors affecting vancomycin’s steady-state trough concentrations were not solely kidney function and dosage. Cholesterol-related lipid indicators and BNP levels at the initiation of vancomycin therapy were also significantly correlated with the steady-state trough concentrations. To facilitate earlier predictions of vancomycin’s steady-state trough concentrations, we tried to develop a machine learning model using clinical indicators collected 48 h prior to the concentration measurement, specifically for forecasting vancomycin’s steady-state trough levels. Among the 546 participants, a random selection of 446 (82%) was used to construct and internally validate the machine learning model, with the remaining 100 (18%) reserved for external validation of the model. By comparing a variety of machine learning models, we found that the random forest algorithm demonstrated the highest accuracy in predicting the steady-state trough concentrations.

**FIGURE 4:**
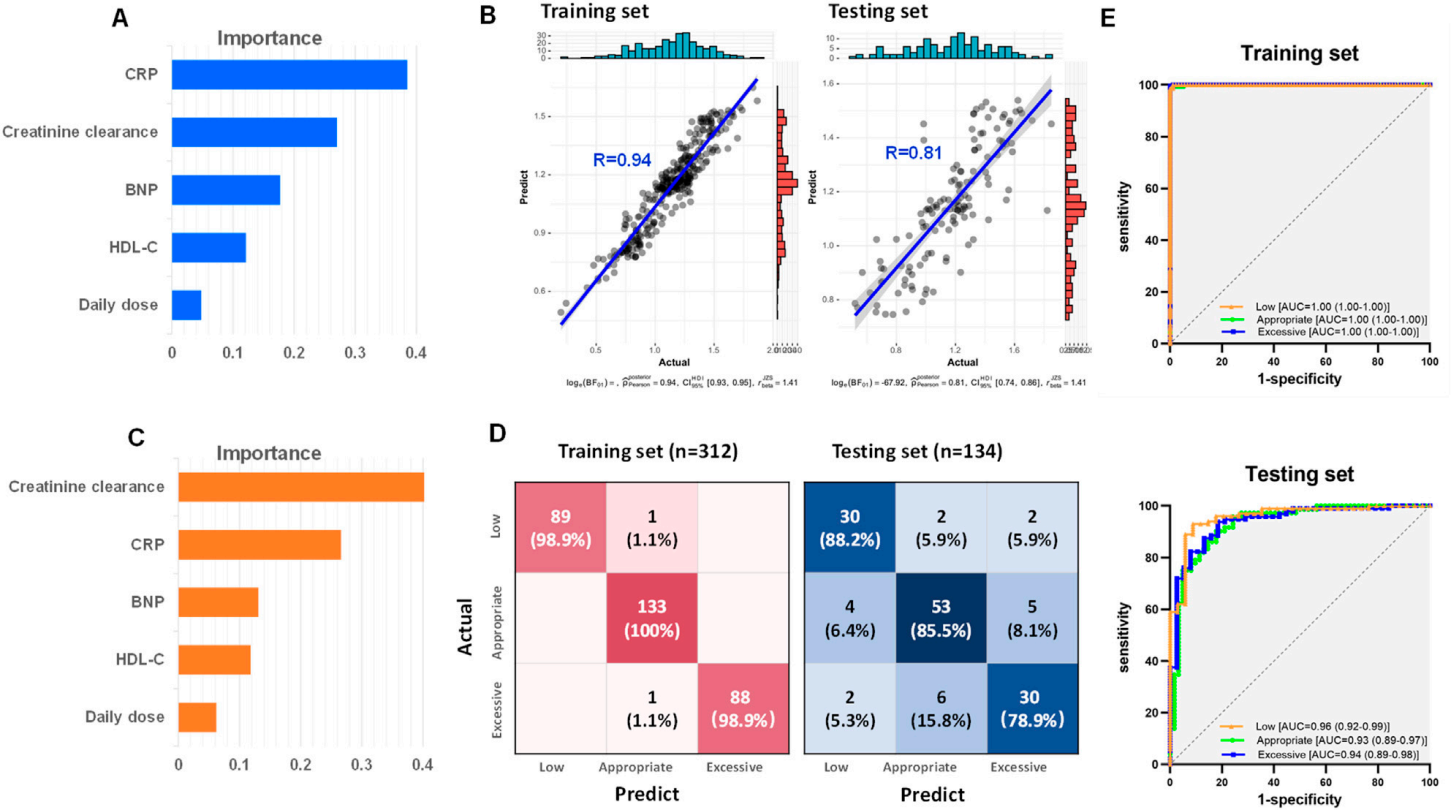
Development of vancomycin steady-state trough concentration prediction model based on creatinine clearance, CRP, BNP, HDL-C and daily dose by random forest. **(A)** Feature importance of the random forest regression model. **(B)** Correlations between the actual and predict steady-state trough concentrations in both training and testing set. **(C)** Feature importance of the random forest classification model. **(D)** Confusion matrix heatmaps for both training set and testing set of random forest classification model. **(E)** ROC curves of both training set and testing set from random forest classification model.

In the development phase of random forest model, all dataset from the 446 subjects were divided into a training set and a testing set in a 7:3 ratio. Creatinine clearance, CRP, BNP, HDL-C and daily dose were selected for the construction of the random forest regression model to predict the steady-state trough concentrations. The feature importance of the regression model was shown in Figure 4A. A comparison between the actual and predicted steady-state trough concentrations of vancomycin in the training set and testing set was illustrated in Figure 4B. The correlation coefficients between the predicted trough concentrations and the actual trough concentrations in the training and test sets were 0.94 and 0.81, respectively (Figure 4B).

To predict whether vancomycin’s steady-state trough concentrations fall within the recommended guidelines, a random forest classification model was further developed based on creatinine clearance, CRP, BNP, HDL-C, and daily dose. The feature importance of the classification model was shown in Figure 4C. This model was designed to distinguish low trough, appropriate trough, and excessive trough groups. The heat maps of the confusion matrix for both the training and testing set were depicted in Figure 4D. The accuracy for the training and testing set were 0.99 and 0.84, respectively. ROC curves and their respective areas under the curve (AUC) for distinguishing between the low, appropriate, and excessive trough groups in both the training and testing set were shown in Figure 4E. The model demonstrated exceptional discrimination capability, with AUC achieving a perfect score of 1 for the training dataset, and maintaining a high level of accuracy with an AUC exceeding 0.93 for the independent testing dataset.

### External validation of random forest models and comparative analysis with Bayesian population pharmacokinetic model

To evaluate the generalization capability and robustness of the machine learning model for predicting vancomycin steady-state trough concentrations, we further conducted external validation of the random forest regression model and the random forest classification model using an additional 100 subjects. To compare the accuracy of the random forest model with the Bayesian PopPK model for predicting vancomycin steady-state trough concentrations, the Bayesian PopPK-based calculator for vancomycin steady-state trough concentrations (https://clincalc.com/vancomycin/) was also used to compute the concentrations for 100 subjects in the external validation set (Kane, 2024).

**FIGURE 5:**
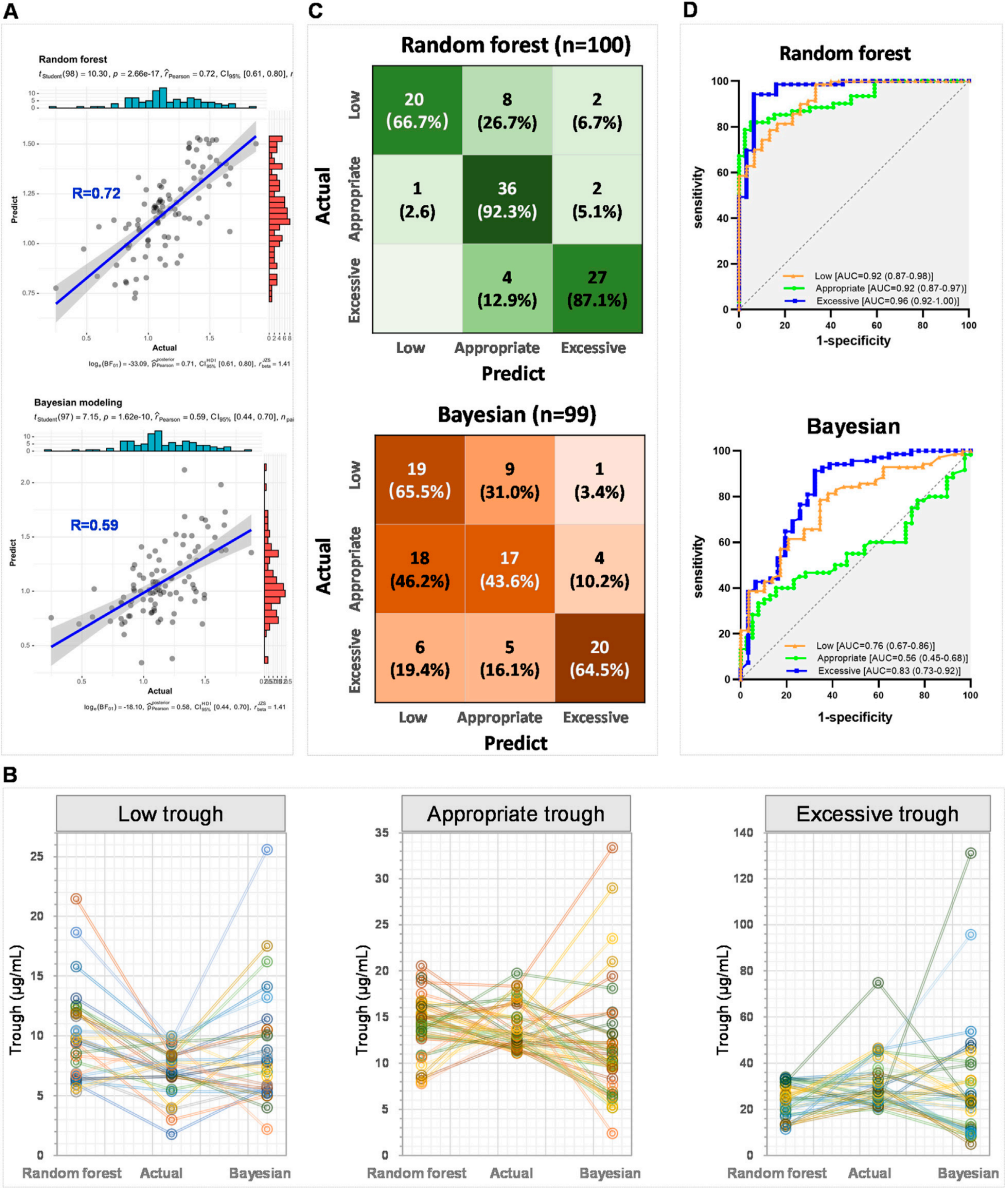
External validation of random forest models and comparative analysis with Bayesian PopPK model. **(A)** The correlation between the actual steady-state trough concentrations and the predict steady-state trough concentrations by the random forest model and the Bayesian PopPK model, respectively. **(B)** Line charts comparing the predicted steady-state trough concentrations by this study’s random forest regression model, the actual steady-state trough concentrations, and the predicted steady-state trough concentrations using the Bayesian PopPK model for each group. **(C)** Confusion matrix heatmaps for random forest model and Bayesian PopPK model. **(D)** ROC curves of random forest classification model and Bayesian PopPK model.

The correlation coefficient between the vancomycin steady-state trough concentrations predicted by the random forest model and the actual steady-state trough concentrations was 0.72 (upper panel of Figure 5A). In contrast, the correlation coefficient between the vancomycin steady-state trough concentrations predicted by the Bayesian PopPK model and the actual trough concentrations was only 0.59 (lower panel of Figure 5A). Figure 5B presented line charts comparing the predicted trough concentrations by this study’s random forest regression model, the actual trough concentrations, and the predicted trough concentrations using the Bayesian PopPK model. It was evident that the random forest model constructed in this study was significantly more accurate in predicting vancomycin trough concentrations than the Bayesian PopPK model, especially for the subjects in appropriate trough group, and excessive trough group.

Subsequently, the accuracy of the random forest classification model and the Bayesian PopPK model in classifying the external validation set was further examined. The random forest model achieved accuracy rates of 66.7%, 92.3%, and 87.1% for the identification of groups low, appropriate, and excessive trough, respectively (upper panel of Figure 5C). In contrast, the Bayesian PopPK model’s accuracy rates for the identification of groups low, appropriate, and excessive trough were 65.5%, 43.6%, and 64.5%, respectively, which were significantly lower than those of the random forest model established in this study (lower panel of Figure 5C). The overall predictive accuracy rates for the random forest model and the Bayesian PopPK model were 0.83 and 0.57, respectively. The random forest model demonstrated impressive AUC values under the ROC curves for low, appropriate, and excessive trough groups in the external validation cohort, with values reaching 0.92, 0.92, and 0.96, respectively (upper panel of Figure 5D). However, the Bayesian PopPK model only achieved areas under the ROC curves of 0.76, 0.56, and 0.83 for low, appropriate, and excessive trough groups in the external validation cohort, respectively (Figure 5D lower panel). By comparing the predictive results of the currently most popular Bayesian PopPK models, the random forest model constructed in this study has a significant advantage in the accuracy of predicting vancomycin steady-state trough concentrations.

## Discussion

Monitoring vancomycin steady-state trough concentrations is vital for optimizing treatment efficacy and safety, preventing resistance, and minimizing nephrotoxicity (Rybak et al., 2020a). However, current clinical methods measure steady-state concentrations around 48 h post-administration, causing delays in treatment adjustments (Prybylski, 2017). This study aims to construct an early prediction model for vancomycin steady-state trough concentrations. We elucidate the complex interplay among factors influencing vancomycin pharmacokinetics and constructs a random forest model for predicting vancomycin’s steady-state blood concentrations by incorporating a broader spectrum of clinical indicators beyond renal function. Random forest is a powerful machine learning method that uses multiple decision trees to make predictions. For regression tasks, it averages the predictions from individual trees, while for classification tasks, it uses majority voting to determine the final outcome. The model introduces randomness by creating bootstrapped subsets of the data and selecting random subsets of features at each split, which helps reduce overfitting and improve robustness. This combination of ensemble learning, randomness, and aggregation makes random forest effective for both predicting continuous values and classifying categorical outcomes. Utilizing the machine learning model developed in this research, clinicians have the capability to evaluate a patient’s prospective steady-state trough concentration prior to the initial detection of steady-state concentration. It enables an early prediction of whether vancomycin’s steady-state trough concentration will fall within the recommended therapeutic range, essentially from the moment treatment commences. This prediction model for vancomycin steady-state trough concentrations offers a practical advantage for clinical decision-making, enabling more precise dosing strategies and enhancing patient safety.

For patients with normal kidney function, the half-life of vancomycin is 6–12 h. According to pharmacokinetic theory, it generally takes about 4-5 half-lives to reach steady-state, which is around 24–48 h. Therefore, for subjects with normal kidney function, the first steady-state trough concentration monitoring (the lowest concentration during the dosing interval) was begin on the third day (48 h after the first administration). For patients with impaired kidney function, the half-life of vancomycin is prolonged; for instance, in patients with acute or chronic renal failure, the half-life of vancomycin can even extend to 8 days (Vandecasteele and De Vriese, 2010). In this study, the time point for the first steady-state trough concentration determination of vancomycin was determined by a senior clinician. Before model construction, we initially screened clinical indicators that affect vancomycin steady-state trough concentrations. A total of 57 different clinical indicators collected from all the 546 subjects within 48 h prior to reaching the steady-state trough concentration of vancomycin were examined for their correlation with first steady-state vancomycin trough concentrations. Statistical results indicated that, in addition to indicators related to kidney function, other clinical indicators such as BNP, cTn I, HDL-C, LDL-C, and platelet, also exhibited significant correlations with vancomycin’s steady-state trough concentrations (Figures 2C,D). All the subjects were further divided into low (<10 μg/mL), appropriate (10–20 μg/mL), and excessive (>20 μg/mL) trough concentration groups, and univariate analysis confirmed statistical differences (P < 0.001) in the mentioned indicators (Table 2), supporting the need for a broader clinical factor consideration in vancomycin trough concentration predication models.

BNP and cTn I are cardiac markers used in clinical practice to assess heart function and detect cardiac conditions (Chapelle, 1999; Hubers et al., 2021). Elevated levels of these biomarkers can indicate heart failure, myocardial infarction, or other cardiac issues. However, there is currently no research that explicitly indicates that BNP and cTn I have a direct impact on the metabolism of vancomycin in the body. The result of the ordinal logistic regression indicated that BNP is a risk factor for exceeding the target steady-state trough concentration of vancomycin, with OR value 1.466 (95%CI: 1.167–1.841) (Figure 3E). Elevated levels of BNP indicate potential heart damage, suggesting that the patient may be experiencing cardiac dysfunction. Impaired heart function can affect blood circulation and the distribution of drugs throughout the body, indirectly impacting vancomycin’s pharmacokinetics and pharmacodynamics (Chuphan et al., 2022). Therefore, in clinical practice, if elevated levels of cardiac enzymes such as BNP are detected in patients, the dose of vancomycin should be adjusted in a timely manner to prevent excessive drug exposure and potential toxic reactions.

Both HDL-C and LDL-C showed a significant negative correlation with vancomycin steady-state trough concentration. Lipoproteins are pivotal for the transport and metabolism of cholesterol in the body, their role in the drug metabolism process, particularly for antibiotics like vancomycin, remains less understood (Yamamoto et al., 2017; Grace, 2012). Further research is warranted to elucidate the complex interplay between lipid metabolism, drug disposition, and the efficacy and safety of vancomycin in various patient populations. The findings in this study highlighted the importance of incorporating patients’ serum HDL-C and LDL-C levels into the dosing strategy for vancomycin. In cases where patients have HDL-C and LDL-C levels below the normal range, it may be necessary to cautiously reduce the dose of vancomycin to decrease the likelihood of toxic effects.

Given the findings of our research that, in addition to renal function, other indicators, such as cardiac biomarkers and lipoprotein also have a significant impact on vancomycin serum concentration, we utilized random forest machine learning models to construct a new predictive model for vancomycin’s steady-state trough concentration. The model incorporated values of creatinine clearance, CRP, BNP, HDL-C, and the daily dose, which were collected 48 h prior to the steady-state concentration measurement. In the random forest regression model, the correlation coefficients between the actual and predicted steady-state trough concentrations of vancomycin for the training and testing sets can reach 0.94 and 0.81, respectively (Figure 4A). The model achieved accuracy rates of 0.99 for the training set and 0.84 for the testing set (Figure 4B), with AUCs of ROC curves reaching 1 for the training set and above 0.93 for the testing set (Figure 4C). The random forest models predicted vancomycin trough levels with high accuracy, factoring in renal function, cardiac biomarkers, lipoproteins, and administration dose.

External validation of our machine learning model has demonstrated its superior predictive accuracy for vancomycin steady-state trough concentrations compared to the traditional Bayesian PopPK model. In the external validation cohort of 100 subjects, the predictive accuracy of the random forest model established in this study reached 0.83, significantly exceeding the predictive accuracy of the Bayesian PopPK model, which was 0.57 (Figure 5). The machine learning model established in this study has improved the accuracy of predicting vancomycin steady-state trough concentrations by 0.26 compared to the Bayesian PopPK model. Compared to Bayesian PopPK models, random forest models have advantages in handling complex nonlinear relationships and high-dimensional data but may require more computational resources and time. Therefore, when indiscriminately including clinical patients, the random forest model established in this study has a significantly higher predictive accuracy for vancomycin’s steady-state trough concentration compared to the Bayesian model.

The random forest model’s good accuracy and high AUC values of ROCs for the classification of trough concentration groups underscore its strength in capturing the nuances of vancomycin pharmacokinetics. The robustness of the random forest model is evident from its consistent performance across the training and testing sets, and its generalization capabilities are supported by the external validation results. These findings suggest that the integration of diverse clinical indicators and advanced machine learning techniques can significantly enhance the predictive power of pharmacokinetic models, potentially leading to improved clinical outcomes through personalized dosing strategies. However, we recognize that our single-center cohort design may introduce geographic bias and result in a relatively homogeneous patient population. This limitation could potentially affect the generalizability of our findings to other regions or patient groups with different demographics, comorbidities, or clinical practices. Therefore, further research is needed to refine the model by incorporating additional research centers and validating model performance in diverse patient populations and clinical settings. Longitudinal follow-up studies are also essential to assess the model’s long-term impact on clinical outcomes, such as treatment efficacy, nephrotoxicity incidence, and patient safety. Additionally, future work should focus on integrating the model into clinical decision support systems to facilitate real-time decision-making and enhance clinical workflow. Overall, ongoing model refinement and validation are crucial for successful clinical translation and broader applicability of machine learning in optimizing antibiotic therapies.

## Data Availability

The original contributions presented in the study are included in the article/supplementary material, further inquiries can be directed to the corresponding authors.
